# Modification of the mean-square error principle to double the convergence speed of a special case of Hopfield neural network used to segment pathological liver color images

**DOI:** 10.1186/1472-6947-4-22

**Published:** 2004-12-12

**Authors:** Rachid Sammouda, Mohamed Sammouda

**Affiliations:** 1 Dept. of Computer Science, University of Sharjah, Sharjah, UAE; 2 Dept. of Computer & Information Science, Prince Sultan University, Riadh, Saudi Arabia

## Abstract

**Background:**

This paper analyzes the effect of the mean-square error principle on the optimization process using a Special Case of Hopfield Neural Network (SCHNN).

**Methods:**

The segmentation of multidimensional medical and colour images can be formulated as an energy function composed of two terms: the sum of squared errors, and a noise term used to avoid the network to be stacked in early local minimum points of the energy landscape.

**Results:**

Here, we show that the sum of weighted error, higher than simple squared error, leads the SCHNN classifier to reach faster a local minimum closer to the global minimum with the assurance of acceptable segmentation results.

**Conclusions:**

The proposed segmentation method is used to segment 20 pathological liver colour images, and is shown to be efficient and very effective to be implemented for use in clinics.

## Background

Segmentation is an important step in most applications that use medical image data. For example, segmentation is a prerequisite for quantification of morphological disease manifestations and for radiation treatment planning [1,2], for construction of anatomical models [3], for definitions of flight paths in virtual endoscopies [4], for content-based retrieval by structure [5], and for volume visualization of individual objects [2].

A Number of algorithms based on approaches such as histogram analysis, regional growth, edge detection and pixel classification have been proposed in other articles of medical image segmentation. In recent years, Artificial Neural Networks (ANNs) have been proposed as an attractive alternative solution to a number of pattern recognition problems. In our previous works [6], we have explored the potential of a Special Case of Hopfield Neural Network (SCHNN) in segmenting cerebral images obtained using the Magnetic Resonance Imaging (MRI) technique.

Hopfield network for the optimization applications consists of many interconnected neuron elements. The network minimizes an energy function of the form:



where N is the number of neurons, *V*_*k *_is the output of the *k*^*th *^neuron, *I*_*k *_is the bias term, and *T*_*kl *_is the interconnection weight between the *k*^*th *^and *l*^*th *^neurons. The energy function used in the segmentation problem is slightly different from the one defined by Hopfield and the arguments are given in [7].

The results that have been obtained in [6] were preferable to those obtained using Boltzmann Machine (BM) and the conventional ISODATA clustering technique. Also, in [8] we have shown that SCHNN is also able to make crisp segmentation of pathological liver colour images. However, during our study attempt to improve the segmentation process, we found that SCHNN segmentation results depend strongly on some parameters in the energy function formulating the classification problem. A summery of this study follows.

## Methods

The segmentation problem of an image of N pixels is formulated in [8] as a partition of the N pixels among M classes, such that the assignment of the pixels minimizes a criterion function. The SCHNN classifier structure consists of a grid of N × M neurons with each row representing a pixel and each column representing a cluster. The network classifies the image of N pixels of P features among M classes, in a way that the assignment of the pixels minimizes the following criterion function:



where *R*_*kl *_is the Mahalanobis distance measure between the *k*^*th *^pixel and the centroid of class *l*, *R*_*kl *_is also equivalent to the error committed when a pixel *k *is assigned to a class *l*. The index *n *in  is the power or weight of the considered error in the energy function of the segmentation problem, and *V*_*kl *_is the output of the *kl*^*th *^neuron. *N*_*kl *_is a N × M vector of independent high frequency white noise source used to avoid the network being trapped in early local minimums. The term *c*(*t*) is a parameter controlling the magnitude of noise which is selected in a way to provide zero as the network reaches convergence. The minimization is achieved by using SCHNN and by solving the motion equations satisfying:



where *U*_*kl *_is the input of the *k*^*th *^neuron, and *μ*(*t*) is a scalar positive function of time, used as heuristically motivated stopping criterion of SCHNN, and is defined as in [6] by:

*β*(*t*) = *t*(*T*_*s *_- *t*)     (4)

where *t *is the iteration step, and *T*_*s *_is the pre-specified convergence time of the network which has been found to be 120 iterations [6]. The network classifies the feature space, without teacher, based on the compactness of each cluster calculated using Mahalanobis distance measure between the *k*^*th *^pixel and the centroid of class *l *given by:



where *X*_*k *_is the P-dimensional feature vector of the *k*^*th *^pixel (here P = 3 with respect to the RGB color space components),  is the P-dimensional centroid vector of class *l*, and Σ_*l *_is the covariance matrix of class *l*. The segmentation algorithm is described as follows [8].

**Step 1 **Initialize the input of the neurons to random values.

**Step 2 **Apply the following input-output relation, establishing the assignment of each pixel to only and only one class.



**Step 3 **Compute the centroid  and the covariance matrix Σ_*l *_of each class *l *as follows:





where *n*_*l *_is the number of pixels in class *l*, and the covariance matrix is then normalized by dividing each of its elements by .

**Step 4 **Update the inputs of each neuron by solving the set of differential equations in (2) using Eulers approximation:



**Step 5 **if *t *<*T*_*s*_, repeats from **Step 2**, else terminated.

**Figure 1:**
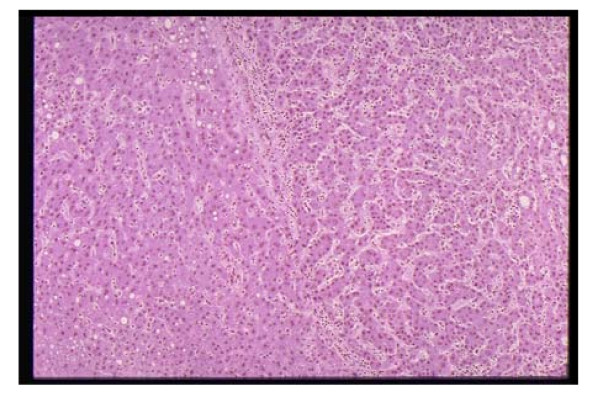
A sample of pathological liver colour image in true colour (Red, Green, and Blue). The cells nuclei are represented by a circular shape in dark purple colour, the cytoplasm regions are coloured purple, the circular objects in white represent the fat cells, and the remaining objects in wave shape and white colour represent the fibrous tissues and blood sinus. The contour of the image is black.

For this study, a total of 20 liver tissue sections were provided by the pathological division of National Cancer Center in Tokyo. These sections were taken using needle biopsy, stained with hematoxylin and then magnified with an optical microscope. Figure 1 shows a true RGB color image of liver tissue of 768 × 512 pixels. We have used the above described SCHNN classifier with the image components in the R.G.B color space. The number of classes is fixed to five based on medical information. These classes are the contour of the image, the cell's nuclei, the cytoplasm, the fibrous tissues, and the class of both blood sinus and fat cells.

**Figure 2:**
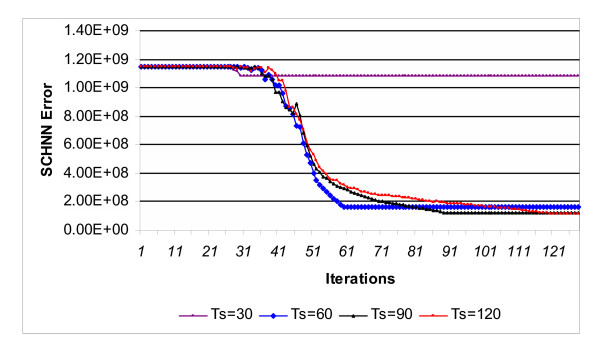
SCHNN energy function curves during the segmentation of the sample shown in Figure 1 using different values of the pre-specified convergence time Ts.

Figure 2 shows the curves of SCHNN energy function during the segmentation of the sample shown in Figure 1 with *T*_*s *_values between 30 and 120 iterations. Similar curves were obtained for the rest of the images of the dataset. As it is illustrated in Figure 2. The curve corresponding to *T*_*s *_= 120 iterations gives the optimal solution, the same as it is with MRI data [6].

In order to study the effect of the weight of the Mahalanobis distance *R*_*kl *_in the cost function (2), we have provided a simple modification to the above algorithm as follows:

**Step 1 **Use the same random initialization N × M matrix, as input of the neurons, when minimizing the energy function (1) with different error's weight *n*.

This condition is added to the algorithm in order to make sure that the random field does not have any effect on the generated results.

**Step 2 **trough **Step 5 **remain the same.

## Results

**Figure 3:**
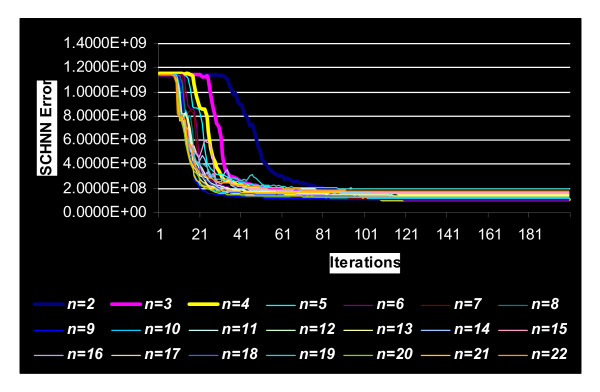
Shows different curves of the optimization of the energy function of the segmentation, of the sample in Figure 1, by considering different values of the variable *n *in equation (2) and with pre-specified convergence Time Ts = 120 iterations.

Figure 3 shows different curves of the optimization of the energy function of the segmentation of the sample shown in Figure 1 using SCHNN with the above modification (Step 1) with respect to different values of the variable *n *in equation (2). As aforementioned, the pre-specified convergence time of SCHNN is fixed to *T*_*s *_= 120 iterations. However, we can clearly see from Figure 3 that with a higher value of *n *in Equation (2), the same convergence point or a close position is reached in half the time of the one reached with *n *= 2 and *T*_*s *_= 120 iterations. So, this raises the following question: what is the type of relation between the variable *n *in (2) and the pre-specified convergence time *T*_*s*_?

**Figure 4:**
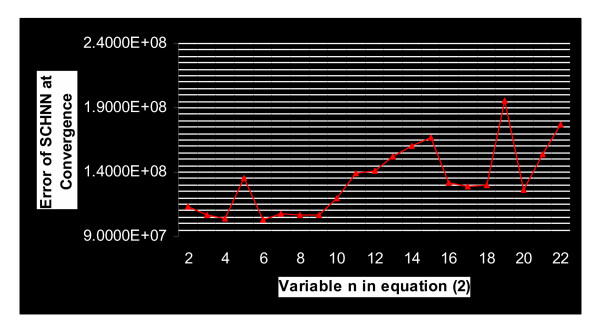
This curve is extracted from Figure 3, it connects the convergence values of the energy function of the segmentation problem, of the sample in Figure 1, by considering different values of the variable *n *in Equation (2), with the same random initialization matrix for all *n *values, and with a pre-specified convergence time Ts = 120 iterations.

Before answering this question, it is essential to know at this level what is the best value of *n *that corresponds to the optimum solution with *T*_*s *_= 120 iterations. From Figure 4, it can be seen that *n *= 6 gives the optimum solution with *T*_*s *_= 120. Similar figures to Figure 3 and Figure 4 were obtained with the rest of the images in the dataset.

## Discussion

### Analysis of the pre-specified convergence time effect

**Figure 5:**
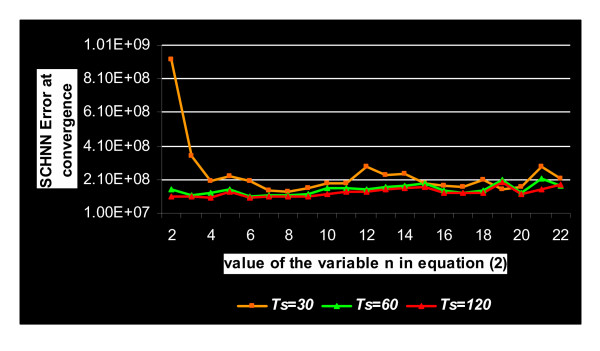
Curves of the energy function of SCHNN at convergence with respect to values of the variable n in Equation (2) and for different pre-specified convergence time Ts. The green and red curves correspond to Ts = 60 and Ts = 120, respectively, are almost identical when n is in the range 5–10.

In order to study the effect of the pre-specified time, we repeated the above experiments with different Ts values. We realized that each value of Ts corresponds to a value of *n*, in Equation (2). When both (*Ts *and *n*) used together they give a local optima in the energy landscape of SCHNN. Figure 5 shows the curves linking the convergence values of SCHNN with respect to the value of *n *in Equation (2) that are obtained with Ts values 120, 60, and 30. We realized that the curves corresponding to Ts = 120 and Ts = 60 intersect in their optimum solutions obtained with *n *= 6, and the two curves are similar when n is in the range 5–10. However, the curve corresponding to Ts = 30, shows higher error at convergence of all values of *n*.

### Analysis of the SCHNN random initialization effect

**Figure 6:**
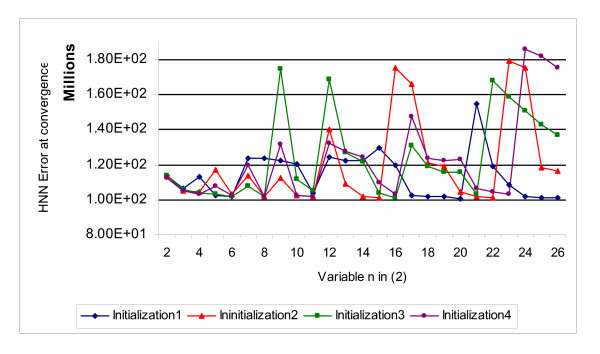
Curves of the energy function of SCHNN at convergence with respect to values of the variable *n *in Equation (2) and for different initialization matrices.

In order to see the effect of the random initialization on the results of the algorithm described in section 3, we have executed the same algorithm with different initialization matrices and the curves of the convergence values of SCHNN corresponding to these initializations are shown in Figure 6. As it is clear from the curves, in Figure 6, the random initialization does not have effect on the variable *n *in (2) when it takes the value of six where SCHNN gives an optimum and acceptable results that agree with the pathological experts point of views. However, with other values of n, the random initialization may affect the solution of the problem, or in other words, may affect the error of the SCHNN at convergence as shown in Figure 6.

## Conclusions

We analyzed the effect of considering the mean-square error in formulating the segmentation problem of multidimensional medical images. We have shown, empirically, that considering an integer power equal to six, of the error in the energy function of the problem, helped SCHNN to converge twice as fast as the same optimal solution obtained with the mean-square error algorithm. This result is promising to make our segmentation method useful for a Computer Aided Diagnosis (CAD) system for liver cancer and the like. In our future work, we will study deeply the effect of the random initialization and its effect on the segmentation result and on the SCHNN classifier.

## Competing interests

The author(s) declare that they have no competing interests.

## Authors' contributions

Rachid carried out the theoretical study, the sequence alignment and drafted the manuscript. Mohammed participated in the design of the study and performed the analysis and helped to draft the manuscript. All authors read and approved the final manuscript.

**Figure 7:**
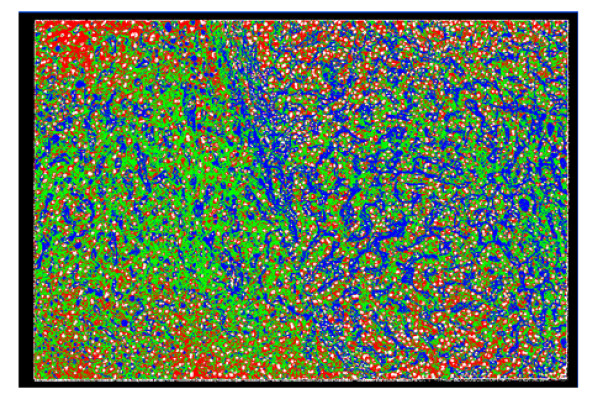
Segmentation result of the sample in Figure 1, obtained using SCHNN in optimizing equation (1) with n = 2, and a pre-specified convergence time Ts = 120 iterations. The cells nuclei are represented by a circulate shape with white colour, surrounded by the red regions representing the cytoplasm of the cells, fat cells are coloured blue, and the fibrous tissues and blood sinus are coloured green.

**Figure 8:**
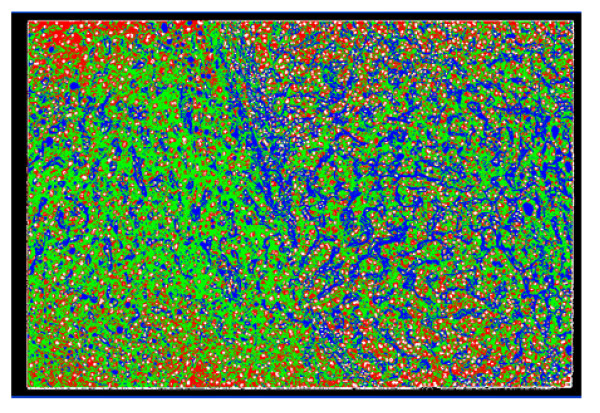
Segmentation result of the sample liver pathological image in Figure 1, obtained using SCHNN in optimizing equation (2) with n = 6, and a pre-specified convergence time Ts = 120 iterations.

**Figure 9:**
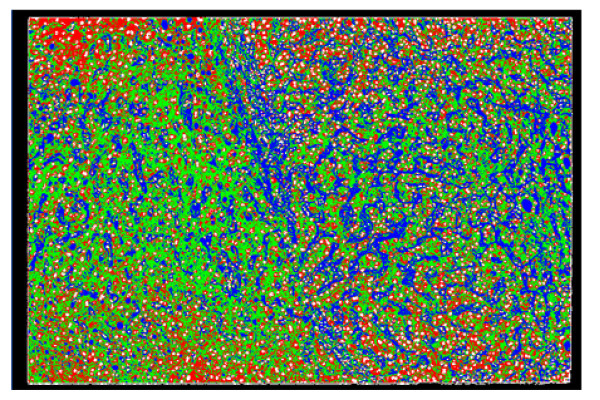
Segmentation result of the sample liver pathological image in Figure 1, obtained using SCHNN in optimizing equation (2) with n = 6, and a pre-specified convergence time Ts = 60 iterations.

**Figure 10:**
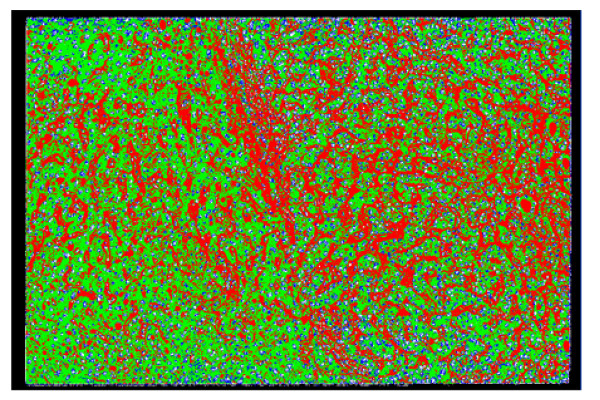
Segmentation result of the sample liver pathological image in Figure 1, obtained using SCHNN in optimizing equation (2) with n = 12, and a pre-specified convergence time Ts = 30 iterations

## Pre-publication history

The pre-publication history for this paper can be accessed here:


